# Percutaneous Transhepatic Bowel Stent Deployment: An Alternative Approach for Malignant Afferent Loop Obstruction Following Whipple’s Procedure

**DOI:** 10.7759/cureus.15964

**Published:** 2021-06-27

**Authors:** Zahid Khan Amin, Raana Kanwal, Atif Nawaz, Alishbah Ziad, Muhammad Shozab

**Affiliations:** 1 Interventional Radiology, Shifa International Hospital, Islamabad, PAK; 2 Diagnostic Radiology, Shifa International Hospital, Islamabad, PAK

**Keywords:** afferent loop syndrome, whipple’s, percutaneous transhepatic metallic stent insertion, aesthetically acceptable, non-surgical possibilities

## Abstract

Afferent loop syndrome is an uncommon postoperative complication. Currently, we lack a therapeutic option for treatment of malignant afferent loop obstruction following procedures like Whipple's. Here we present a case of afferent loop obstruction in a known case of pancreatic carcinoma, status after Whipple’s procedure, in which we used a percutaneous transhepatic approach to relieve the afferent loop obstruction using a self-expanding bare metal stent.

## Introduction

One of the complications of a pancreaticoduodenectomy, also known as Whipple’s procedure, is afferent loop obstruction, which can be fatal if the patient develops jaundice or cholangitis [1]. Procedures like Whipple’s, Billroth-II gastrojejunostomy (B-II) and Roux-en-Y gastroenterotomy (R-Y) have a rare complication of afferent loop syndrome [2]. Afferent loop syndrome occurs as a result of partial or complete obstruction of the afferent limb along its course or at the anastomosis. Symptoms are related to both the distention of the bowel as secretions accumulate and the obstruction of the pancreatico-biliary tree [3]. Up to 1% of patients who undergo partial gastrectomy with Billroth II or Roux-en-Y reconstruction will experience afferent loop syndrome [4]. For benign causes, surgery is the mainstay of treatment, whereas, in the setting of malignancy, neo-adjuvant therapy often followed by palliative surgery is the standard management.

## Case presentation

**Figure 1:**
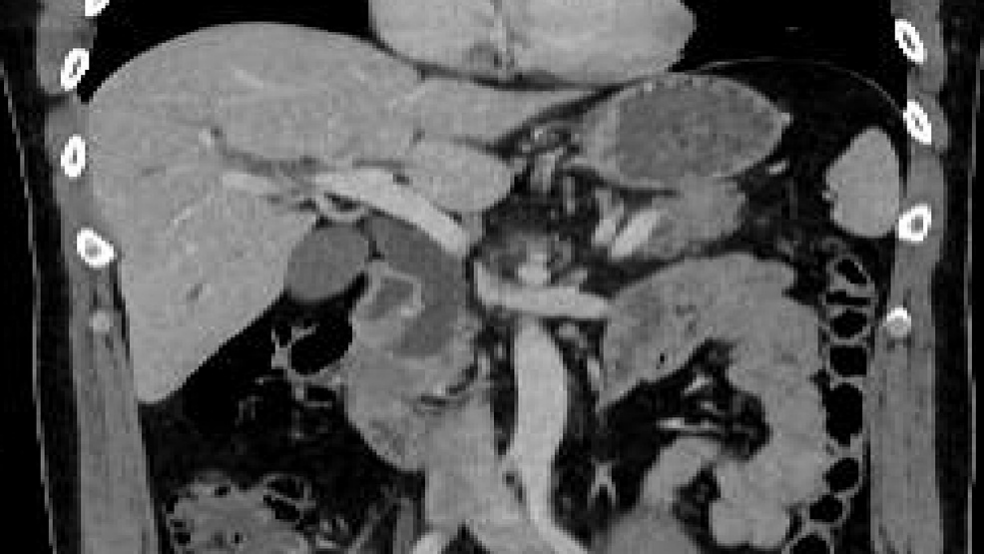
Soft tissue density mass lesion in the periampullary region with upstream moderate intra- and extrahepatic biliary dilatation.

A 47-year-old male patient presented to the ER with a complaint of jaundice. An ultrasound was performed in an outside facility, which showed a pancreatic mass. CT scan of the abdomen and pelvis was done, which revealed a soft tissue density mass lesion in the periampullary region with upstream moderate intra- and extrahepatic biliary as well as pancreatic duct dilatation (Figure 1).

**Figure 2:**
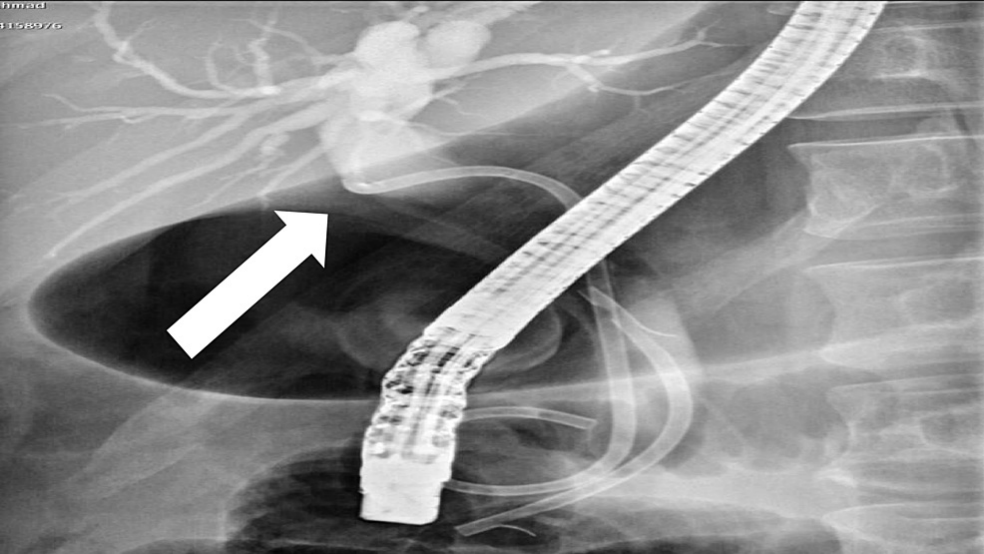
Endoscopic retrograde cholangiopancreatography images showing common bile duct and pancreatic duct stent placements.

Endoscopic retrograde cholangiopancreatography was performed in which common bile duct (CBD) and pancreatic duct stent were deployed (Figure 2).

**Figure 3:**
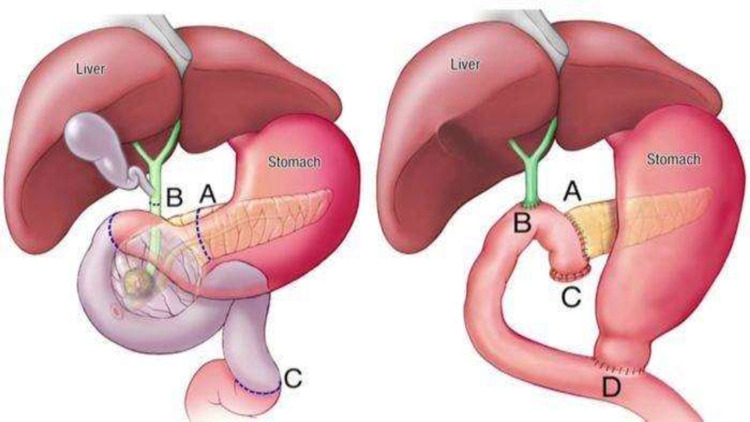
Illustrates the Whipple’s procedure.

The patient subsequently underwent a Whipple’s procedure, and biopsy showed moderately differentiated periampullary adenocarcinoma. Subsequently, chemotherapy was started and the patient was discharged (Figure 3).

**Figure 4:**
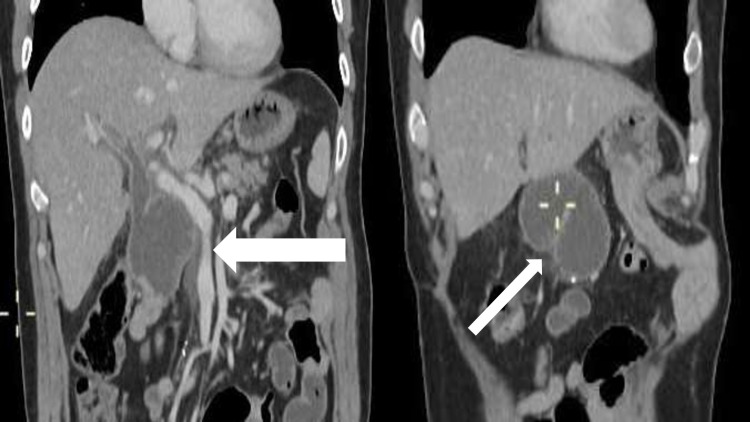
Showing enhancing circumferential wall thickening in the distal part of pancreaticojejunostomy loop resulting in upstream dilatation of the afferent loop and dilation of intrahepatic biliary channels.

A few months later, patient presented to the ER with complaints of fever, pruritus, and yellow skin discoloration, suggestive of obstructive jaundice and biliary sepsis. The patient had a CT abdomen performed, which showed evidence of local recurrence with an ill-defined, focal soft tissue mass obstructing the afferent loop several centimeters distal to CBD resulting in upstream dilatation of the afferent loop as well as that of intrahepatic biliary channels. Adjacent ipsilateral ureter was also involved with resultant hydronephrosis (Figure 4).​​​​​​

**Figure 5:**
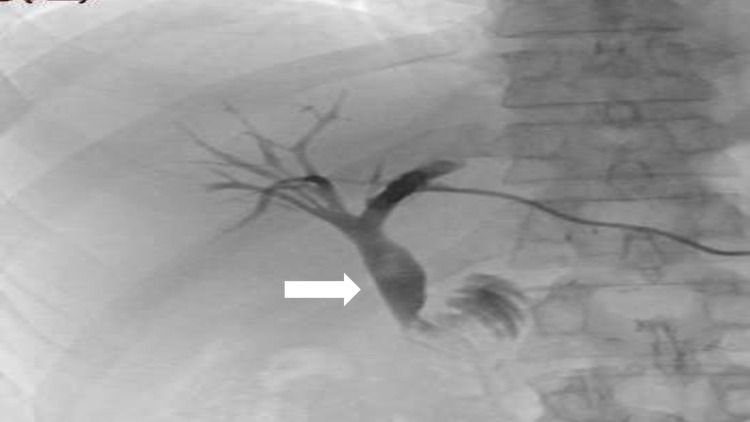
Cholangiogram showing biliary dilatation.

Due to the post-Whipple anatomy, endoscopic biliary drainage was not possible; therefore, the patient was referred to the interventional radiology department for percutaneous biliary drain placement. Initially, an external percutaneous transhepatic biliary drain was placed from the left lobe of the liver (Figure 5).

The patient otherwise was relatively well with a good performance status, i.e. performance status 1 according to Eastern Cooperative Oncology Group, but was not happy with the esthetic appearances and the care needed in looking after the attached external drainage bag. He was very keen if some procedure could be done to enable internal drainage. This would also allow for prevention of the physiological loss of bile salts and pancreatic secretions as well as improve his digestion. Therefore, the case of the patient was discussed again with the interventional radiologist to see if any further intervention was possible when he attended for a follow-up visit. 

Following review of his images it was decided that if the malignant obstruction in the afferent loop could be traversed, then a self-expanding metallic stent could be deployed to enable internal drainage but this would need to be done via a transhepatic approach. 

**Figure 6:**
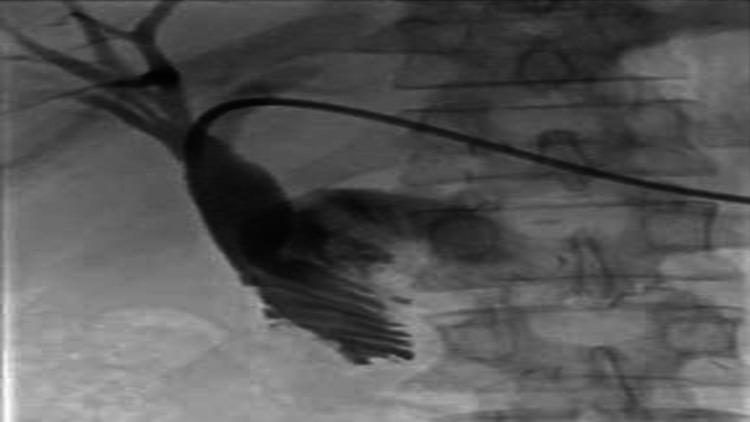
Abrupt cutoff of contrast due to tumor infiltration in the afferent loop.

He was admitted electively for this procedure. The patient already had an external biliary drain in situ, which had recently been placed during his current admission to decompress the biliary system. The drain was removed and replaced with a sheath followed by catheter placement to visualize the roux loop and site of obstruction, which was noted to be proximal to the jejunojejunal anastomosis (Figure 6).

**Figure 7:**
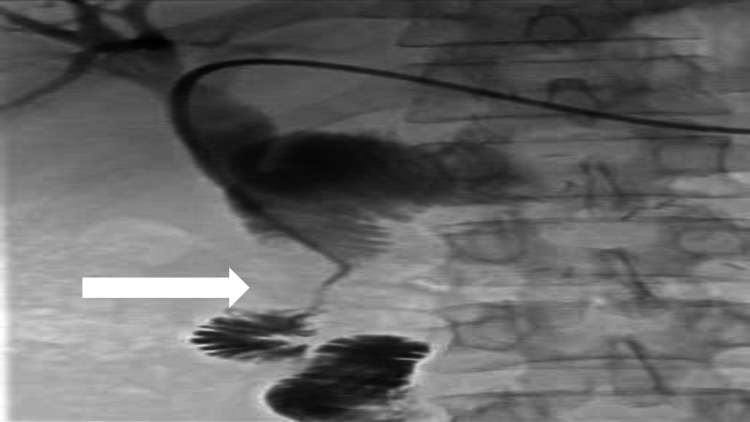
Narrow lumen demonstrated.

This stricture was negotiated with a guidewire and a 5-Fr angle tip catheter was advanced into distal jejunal loop. A short segment of narrowed bowel lumen due to tumor infiltration was demonstrated with contrast injection while pulling the catheter back (Figure 7).

**Figure 8:**
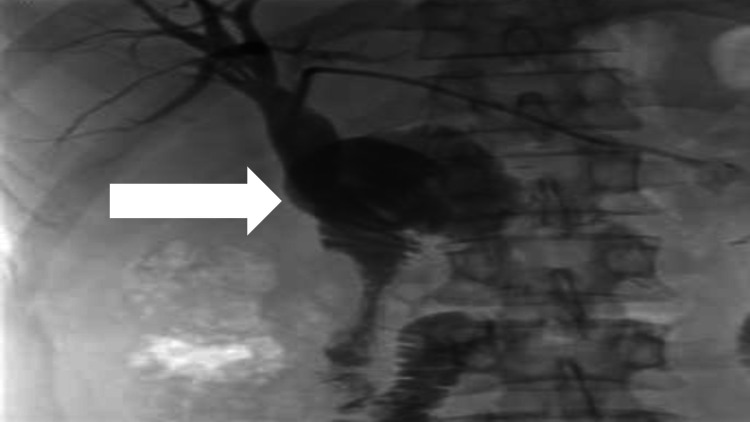
Good flow of contrast seen through the stent finally.

The stricture was crossed with the guidewire again and exchanged for a stiff Amplatz wire via the angle tip catheter. Subsequently, a self-expandable metallic stent (Epic - Boston Scientific) 14 x 60 mm was deployed. Stent was dilated with a 8 x 40 mm (RIVAL PTA) balloon. An hourglass appearance of the stent was produced to prevent stent migration. Finally, good contrast flow through the stent was noted, showing adequate luminal dilatation (Figure 8).

Subsequently, an 8.5-Fr locking pigtail external drainage catheter was placed in the blind end of the loop and capped. No immediate or late complications were seen. The patient remained vitally stable and was able to tolerate oral feed. A urological consultation was also made prior to the interventional procedure and during the same admission it was also possible to place a ureteric stent, thus alleviating his hydronephrosis and avoiding the need of a nephrostomy tube.

A year after the procedure, the patient was called, through the given demographic contact details, and he was duly satisfied. No adverse outcome or complication was reported.

## Discussion

Procedures like Whipple’s, B-II, and R-Y have afferent loop syndrome as an uncommon but a serious complication. Patients with the complication of afferent loop syndrome present with a range of symptoms including abdominal pain, nausea, vomiting with resultant malnutrition and weight loss, jaundice, and fever [5]. Dilatation of the afferent bowel loop and the resultant biliary dilatation is likely due to accumulation of biliary, pancreatic, and intestinal secretion secondary to bowel loop obstruction. Afferent loop obstruction can occur due to benign and malignant causes. Most of the causes are benign, including kinking at the anastomotic site, internal herniation, or inflammation surrounding the anastomosis; however, the malignant causes are rare [6].

Treatment options for malignant afferent loop obstruction include surgical as well as non-surgical options. Although surgery is effective, non-surgical options are desirable due to the presence of adhesions, especially in the setting of metastatic disease, where the goal is to improve the patient’s quality of life and not the overall survival [7]. 

Non-surgical possibilities include endoscopic approach to afferent loop; however, change in anatomy following surgery makes this option difficult. The second option is direct percutaneous puncture of the afferent loop if the loop is adjacent to the anterior abdominal wall but peritonitis is a known complication of this option. Another option that could be sought of includes a transhepatic route that is a fair option, but in cases where there is no intrahepatic biliary dilatation, transhepatic approach would be difficult.

## Conclusions

Interventional radiology can be a ray of light in the darkness by providing a safe and an efficient alternative for afferent loop obstruction other than endoscopic or surgical options. Thus, percutaneous transhepatic metallic stent deployment for malignant bowel stricture is a preferable alternative treatment option.
